# Application Of The American College Of Emergency Physicians (ACEP) Recommendations And a Risk Stratification Score (OESIL) For Patients With Syncope Admitted From The Emergency Department

**Published:** 2011-10-02

**Authors:** Adrian Baranchuk, William McIntyre, William Harper, Carlos A Morillo

**Affiliations:** 1 Queen's University, Arrhythmia Service, Kingston, ON, Canada; 2 McMaster University, Department of Medicine, Hamilton, ON, Canada

**Keywords:** Syncope, ACEP recommendations, OESIL score

## Abstract

**Aims:**

The goals of this study were to apply the 2001 ACEP recommendations for admission to hospital after a syncopal event and to validate the OESIL risk stratification score, in patients with syncope admitted to a general internal medicine ward.

**Methods:**

A retrospective study applied the 2001 ACEP recommendations and OESIL score to all the patients admitted from the emergency department to a general internal medicine ward with a diagnosis of syncope during a 12-month period. The patients were classified as meeting criteria for 2001 ACEP class B or C recommendations and OESIL score 0-1 (low-risk for a major cardiac event) or 2-4 (high-risk for a major cardiac event). The sensitivity and specificity of each group for predicting high-risk patients was calculated.

**Results:**

After applying the 2001 ACEP recommendations to our population, 25% (19 patients) were classified as level B, whereas 68% of the patients were classified as Level C. Sensitivity for ACEP level B recommendations was 100% and specificity was 81%. The ACEP level C recommendations also had 100% sensitivity but markedly reduced specificity at 26%. An OESIL score of 0-1 points was calculated for 30.6% of the population, identifying them as low-risk. An OESIL score of 2-4 points was documented in the remaining 69.4% with a mortality risk of 20 % /year.

**Conclusion:**

A significant proportion (30%) of patients presenting with syncope to a tertiary care University Hospital emergency department and admitted to an Internal Medicine ward were retrospectively classified as low-risk and could have potentially been managed as outpatients. Implementing current guidelines and clinical pathways for the management of syncope may improve this approach.

## Introduction

Syncope is a common medical problem that accounts for 1% to 1.5 % of emergency department (ED) visits [1] and up to 6 % of hospital admissions. Data from the National Hospital Ambulatory Medical Care Survey (NHAMCS) analyzed for the period 1992-2000 showed that over a total of 865 million visits to the ED, 6.7 million (0.77%; 95% CI=0.69-0.85%) were syncope related [2].

Several guidelines, scores and recommendations for the diagnosis and management of patients with syncope have been published in the last decade [3-9]. Some provide recommendations on the need for admission and further diagnostic workup to rule out high-risk causes of syncope; however it remains a reality that a significant number of low-risk patients are unnecessarily admitted for further investigation.

The American College of Emergency Physicians (ACEP) [1,10] has developed and revised a clinical policy for physicians in the evaluation and management of patients presenting to the ED with syncope (Table 1).

Establishing risk is essential in the diagnostic approach to the patient with syncope. For this purpose, The Osservatorio Epidemiologico sulla Sincope nel Lazio (OESIL) [7] developed and validated a simple risk stratification score for patients presenting to the ED with syncope (Table 2). Appropriate identification of high-risk sub-groups of patients in the ED may be beneficial by avoiding unnecessary admissions [8].

The most frequent cause of syncope presenting to either outpatient clinics or the ED remains reflex (neurally mediated) syncope [9]. However, patients visiting the ED frequently undergo several high cost and low-yield diagnostic investigations in order to rule out cardiac and neurological causes of transient loss of consciousness. This is done despite the fact that the risk of sudden death in this group is considerably low [11,12].

The goals of this study were: (1) to retrospectively apply the 2001 ACEP recommendations for admission after a syncopal event and (2) to validate the OESIL risk stratification score, in patients with syncope admitted to a general internal medicine ward.

## Methods

A retrospective search was conducted by reviewing clinical records of all the patients admitted from the ED with a diagnosis of syncope during a 12-month period to the general internal medicine ward. Two blinded investigators (AB, CAM) reviewed all the electronic charts and retrospectively applied the 2001 ACEP recommendations [10] and OESIL score [7].  Disagreements among observers were solved by consensus. Follow-up was conducted by reviewing the same electronic chart for a mean follow-up of 12.5 ± 2.5 months. This study was approved by the Research Ethics Board of McMaster University, HHSC.

### Definitions

Syncope was defined as a transient loss of consciousness due to transient global cerebral hypoperfusion characterized by rapid onset, short duration, and spontaneous complete recovery [9].

Patients were included in the analysis if the admission diagnosis was syncope. The final diagnosis was assigned on the basis of accepted diagnostic criteria for reflex syncope (neurally mediated syncope), orthostatic hypotension and orthostatic intolerance syndromes or cardiac syncope (cardiovascular) [9].

The variables included for analysis were: (1) demographic data, including age and gender; (2) previous episodes of syncope; (3) past medical history; (4) clinical characteristics such as emotional distress prior to the episode, concomitant palpitations, chest pain, dyspnea, focal neurological abnormalities; (5) prodromal symptoms such as lightheadedness, nausea, diaphoresis, weakness, and visual disturbances; (6) medications; (7) physical examination. Recurrence of syncope and major cardiovascular events (including death) were reviewed during the follow-up period.

Coronary artery disease was considered if a history of myocardial infarction or coronary revascularization procedures was documented. Valvular disease was considered if mitral or aortic stenosis or regurgitation were documented by clinical examination and quantified by Doppler. Valvular replacements were also included.

ECGs were classified as normal, sinus bradycardia or arrest, second and third degree AV block, atrial flutter and fibrillation, ventricular tachycardia, conduction disturbances (LBBB/RBBB), long or short QT, pacemaker or ICD malfunction, and acute ST changes. The etiology of syncope was classified according to the European Society of Cardiology Guidelines for the diagnosis and management of syncope (version 2009) [9] (Table 3), as well as "Neurologic" syncope: This category is intended to account for conditions that were initially incorrectly diagnosed as syncope. This category includes seizures, metabolic disorders, vertebrobasilar ischemia, and cataplexy, drop attacks, falls, transient ischemic attacks of carotid origin and psychoghenic pseudosyncope.

### Data Analysis

The patients were classified as meeting criteria for 2001 ACEP class B or C recommendations and OESIL risk score 0-1 (low-risk for a major cardiac event) or 2-4 (high-risk for a major cardiac event). The sensitivity and specificity of each group for predicting high-risk patients was calculated.   Results are expressed as means + standard deviation. Kaplan-Meier survival curves were generated based on OESIL syncope score for patients admitted from the ED with syncope.

## Results

The demographic features of the study population are summarized in Table 4. During a 12-month period, 75 patients over the age of 18 were admitted to the internal medicine ward of the Hamilton General Hospital with an initial diagnosis of syncope. Patients with presyncope, vertigo or dizziness were excluded. The mean age was 68 ± 14 years, 41 % were female. Structural heart disease was present in 60 % and an ECG was abnormal in 16% (Table 4).

After complete evaluation in the ED and hospital admission, a final diagnosis of syncope was attained in 40 patients (54 %), of which 22 were classified as reflex (neurally mediated) syncope (55 %), 6 were cardiac (15%), 10 had orthostatic hypotension (25 %) and 2 had "neurologic" syncope (5 %).

Among patients with cardiac causes of syncope, 2 had ventricular tachycardia, 3 had bradyarrhythmias (2 severe sinus bradycardia, 1 complete heart block) and 1 had an acute coronary syndrome. Among patients with reflex syncope, the diagnosis was made based on clinical history, ECG and physical examination in 18 patients and with a tilt table test in another 4 patients. A history of situational syncope was documented in 7 patients.  No carotid sinus hypersensitivity was found in this cohort.

Only 4 % of patients were taking antiarrhythmic medications at the time of admission and 58.6 % were taking antihypertensive medications. The average in-hospital length of stay was 4.2 ± 3.7 days.

### 2001 ACEP Recommendations

**Figure 1:**
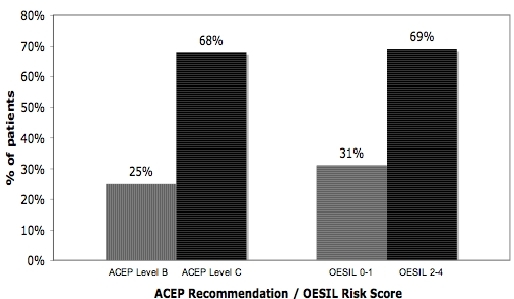
Percentages of patients admitted from the emergency department according to calculated 2001 ACEP recommendation and OESIL risk score

When the 2001 ACEP recommendations were applied to our population, 25% of the patients satisfied at least one of the criteria for level B (Figure 1): 16% had an ECG showing ischemia, arrhythmia or bundle branch block; 4% had previous history of ventricular arrhythmias, 1.5% presented as an acute coronary syndrome and 9% had history of congestive heart failure or evidence of valvular disease on physical examination.

With respect to level C recommendations, 68% of the patients met at least one criterion, without meeting Level B criteria (Figure 1), of which 76% were greater than 60 years of age.

Sensitivity for ACEP level B recommendation was 100% (all patients with a final diagnosis of cardiac syncope were identified by using these recommendations) and specificity was 81%. The ACEP level C recommendations also had 100 % sensitivity but specificity was markedly reduced at 26%.

### OESIL Risk Score

An OESIL risk score of 0-1 points was calculated for 30.6% of the population, identifying them as low-risk. Age older than 65 years was the most frequently encountered clinical predictor (68%). An OESIL score of 2-4 points was documented in the remaining 69.4%, with a mortality risk of 20 % per year (Figure 1).

### Follow-up

**Figure 2:**
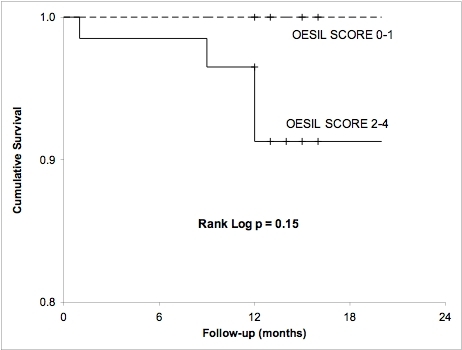
Kaplan-Meier survival curves by OESIL syncope score for patients admitted from the ED with syncope

The mean follow-up (± SD) was 12.5 ± 2.5 months. During follow-up, 14 patients (18.6%) had a recurrence of syncope that required hospital admission. Six patients (8%) died during the follow-up of which two thirds (4/6) were initially categorized as having ACEP recommendations Level B for admission. All of them were identified as high-risk patients (2-4 points) according to the OESIL score. The Kaplan-Meier survival curve is shown in Figure 2. All the deaths occurred within the group of patients with OESIL score of 2-4, and all occurred during the post-admission follow-up period.

## Discussion

The main finding of this retrospective study was that a significant proportion of patients presenting with presumed syncope to a tertiary care ED, and admitted to an Internal Medicine ward were retrospectively classified as low-risk by ACEP and OESIL scores and could have potentially been managed as outpatients or through an urgent out-patient syncope clinic.

### 2001 ACEP Recommendations

The application of level B ACEP recommendations showed a sensitivity and specificity of 100% and 81%, respectively for detection of cardiac syncope. Application of the 2001 ACEP recommendations identified all patients with cardiac syncope requiring admission. Similarly, a reasonable number of patients with low-risk syncope at the ED that could have been managed as outpatients were also identified. Elesber and colleagues [12] reported consistent results when the 2001 ACEP level B recommendations were retrospectively applied to a population of 115 patients admitted from the ED (100% sensitivity, 95% CI 86-100 %; 81% specificity, 95% CI 75-87 %).

Applying level C recommendations maintains high sensitivity (100%) at the cost of markedly reducing specificity (26%) leading to a significant proportion of unnecessary hospital admissions of low-risk patients. This is also concordant with Elesber's findings (100% sensitivity, 95 % CI 86-100 %; 33% specificity, 95% CI 26-40 %) [12]. The limitations of the level C recommendations has lead the ACEP committee to revise these in 2007, and removed them altogether, confirming the lack of specificity as also documented in the present study [1].

Several differences exist between the 2007 and 2001 Level B Recommendations.  The first level B recommendation is that patients with syncope and evidence of heart failure or structural heart disease should be admitted. The second level B recommendation advises admitting patient with syncope and other factors that lead to classification as high-risk for adverse outcome.  These factors, some of which were Level C in 2001, include age and comorbidities, abnormal ECG, hematocrit below 30, and a history of heart failure, coronary artery disease or structural heart disease.

Two recommendations that were part of the 2001 ACEP recommendations have been dropped altogether as indications for admission; family history of sudden unexpected death, and exertional syncope in younger patients without an obvious benign etiology. Age is also no longer treated as a discrete variable, but is believed more to be a reflection of the increased burden of co-morbidities that goes with age.  The updated guidelines would be unlikely to significantly affect our study given that the changes are minor.  Overall, removal of level C recommendations seems to be favorable in that they are unlikely to identify any high-risk patients missed by Level B recommendations, and only add low-risk patients.

In another retrospective analysis, Crane [13] divided a population with syncope in the ED according to the ACEP recommendations into three groups (absolute indication for admission, probable indication and no indication for admission). Thirty six per cent of the absolute indication for admission group died over a 12-month follow-up period compared to none from non-admitted group (p=0.01).

This study, like ours, highlights that the 2001 ACEP and OESIL recommendations are successful in identifying high-risk patients.  The problem remains in identifying low risk patients who can be investigated on an out-patient basis.

### OESIL Risk Score

The OESIL score is a simple risk stratification score for patients with syncope in the ED. This score was validated in a cohort of 270 patients from six community hospitals in Italy. Overall mortality was strongly correlated with the score. Patients with a score 0-1 points had less than 1% mortality at 1 year follow-up, 2 points 19.6 %, 3 points 34.7 % and 4 points 57.1 % (p=0.0001) [7]. This study identified clinical and electrocardiographic variables that could be used in the ED to identify patients with higher-risk syncope.

We considered that a 20% mortality risk at one year was high and merits classification of these patients as high-risk. Our population was divided into two groups: 0-1 points (low-risk, mortality less than 1%) and 2-4 (high-risk, mortality > 20%). More than 30% of the patients admitted had a score of 1 or less suggesting these patients were low-risk, nevertheless they were admitted suggesting a lack of a standardized approach to determine hospital admission at our ED potentially leading to unnecessary admissions. Seventy percent were originally classified in the ED as high-risk patients, and this is likely an overestimation based on the categories designed by the OESIL risk score. Of note, overall mortality (8 %) was considerably lower in comparison to 0.8 %, 19.6 %, 34.7%; 57.1% in the OESIL study groups.  This could be explained by the fact that follow-up was through electronic charts, and thus patients not originally admitted were lost to follow-up. Also, we considered OESIL scores of 2-4 in a single high-risk group and mortality could tend towards the lower end of that group.

### Diagnostic yield, length of stay and Syncope Unit

Disertori and coworkers (EGSYS) [14] conducted a prospective observational registry that included 996 patients with syncope over a total of 105,173 ED patient visits in a one-month period.  The mean in-hospital stay for these patients admitted with syncope was 8.1 ± 5.9 days, primarily in the internal medicine ward. In our series, the mean length of stay was 4.2 ± 3.7 days but the diagnostic yield was considerably lower than in the EGSYS registry (54 % vs. 80%, respectively). This difference may be explained by the fact that EGSYS was a multicenter study using dedicated Syncope Units while in our hospital patients were admitted from the general ED without a standardized approach.

In the SEEDS trial [15] the availability of a 24/7 Syncope Unit in the ED improved the diagnostic yield from 10% in the standard care approach compared to 67% in patients managed by the Syncope Unit (p<0.001). Admissions were reduced from 98% to 43% (p<0.001) and total patient-hospital days were reduced from 140 to 64.

These observations are in agreement with a retrospective analysis by our group applying an algorithmic diagnostic approach to patients presenting with syncope to the ED that showed an increase in diagnostic accuracy from 15% to 77% [8]. It is also concordant with a recent multicentre prospective observational study that was carried out in 19 Spanish hospitals over a one-month period [16]. Adherence to ESC guidelines for syncope management was low and many diagnostic tests were performed with low diagnostic yield (0-12%). In the ED, 1217 (86%) patients received a final diagnosis of syncope, whereas the remaining 202 (14%) were diagnosed with non-syncopal transient loss of consciousness (NST-LOC). After final review, 1080 patients (76%) were diagnosed with syncope, whereas 339 (24%) were diagnosed as NST-LOC (P < 0.001). [16]. Recent studies clearly indicate that improvement of diagnostic yield and proper risk stratification of patients presenting with syncope to the ED can be achieved by knowledge translation interventions and enforcement of guidelines. [17,18]

## Study limitations

This study has several limitations. Retrospective analysis was done by reviewing electronic charts and our interpretation of the cause of syncope is thus limited. As many other patients with potential syncope were not admitted, the calculation of sensitivity was done on this selected population that could therefore be inherently biased. Follow-up was conducted by reviewing the electronic charts and some of the recurrences in non-admitted patients and the out-of-hospital deaths were not recorded in the chart and may have been missed underestimating the overall mortality risk. It also needs to be considered that the ACEP and OESIL scores were not weighted scores and all the variables were assigned the same value (i.e., 1 each). Nonetheless our results are in keeping with prospective studies with the exception of the mortality derived from the OESIL score, suggesting that the above limitations may have a limited effect on the validity of our results.

## Conclusions

A significant proportion (30%) of patients presenting with syncope at a tertiary care University Hospital ED admitted to an Internal Medicine ward were retrospectively classified as low-risk and could have potentially been managed as outpatients. ACEP level B recommendations had an excellent sensitivity (100%) identifying cardiac causes of syncope, however overestimating cardiac causes leading to unnecessary admissions. Broader recommendations (Level C) have poor specificity (26%) also leading to unnecessary admissions.

The OESIL score also identified 30 % of the admitted patients as having very low 1-year mortality (<1 %) potentially also having been managed as outpatients. The systematic application of ACEP (Level B) recommendations and OESIL score to patients presenting with syncope at the ED may be useful in reducing unnecessary hospital admissions and identifying high-risk cardiac patients with syncope.

## Figures and Tables

**Table 1:**
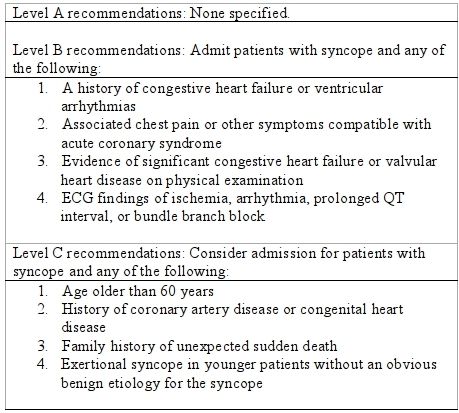
ACEP 2001 Patient management recommendations: admission after a syncopal event

**Table 2:**
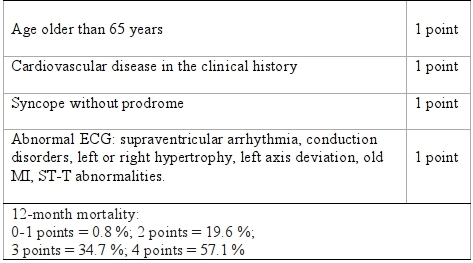
OESIL risk stratification system for patients with syncope in the emergency department

**Table 3:**
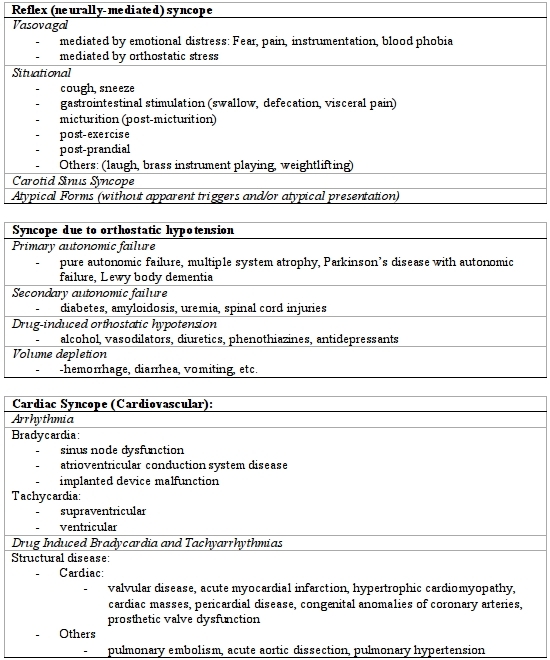
ESC 2009 Classification of Syncope

**Table 4:**
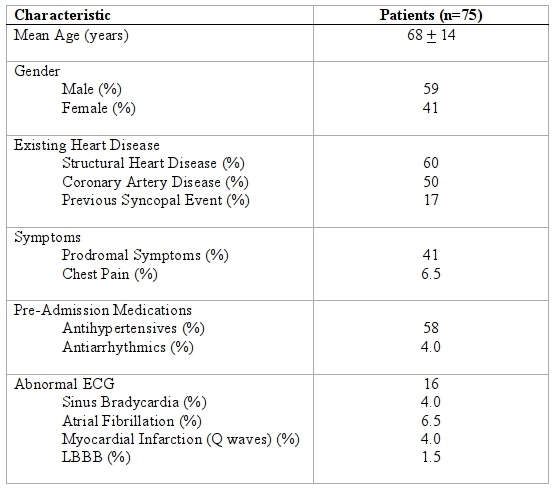
Clinical characteristics of patients admitted to the Internal Medicine Service with an Emergency Department Diagnosis of syncope
